# Associations of tumor necrosis factor alpha genetic variants with metabolic syndrome and type 2 diabetes mellitus in a Thai population

**DOI:** 10.1371/journal.pone.0346147

**Published:** 2026-04-02

**Authors:** Metha Yaikwawong, Khanittha Kamdee, Kasarnchon Mek-yong, Somlak Chuengsamarn

**Affiliations:** 1 Department of Pharmacology, Faculty of Medicine Siriraj Hospital, Mahidol University, Bangkok, Thailand; 2 Division of Endocrinology and Metabolism, Faculty of Medicine, HRH Princess Maha Chakri Sirindhorn Medical Center, Srinakharinwirot University, Nakhon Nayok, Thailand; Universiti Sains Malaysia, MALAYSIA

## Abstract

This work aimed to clarify how polymorphisms in the *TNF* gene relate to metabolic syndrome (MetS), type 2 diabetes mellitus (T2DM), and a broad spectrum of cardiometabolic characteristics, while also determining their impact on circulating TNF‑α concentrations. A total of 765 participants were genotyped for rs1800629 and rs361525, and serum TNF-α was also measured. To assess these relationships, multivariable logistic regression models—incorporating age, sex, and body mass index (BMI)—were applied to estimate adjusted odds ratios (aORs) and their corresponding 95% confidence intervals (CIs). Both variants were significantly associated with MetS: rs1800629 (crude OR = 2.22, 95% CI: 1.45–3.44, *P* < 0.001; adjusted OR = 3.13, 95% CI: 1.78–5.06, *P* < 0.001) and rs361525 (crude OR = 2.08, 95% CI: 1.07–4.21, *P* = 0.035; adjusted OR = 2.84, 95% CI: 1.17–7.31, *P* = 0.025). The rs1800629 variant was also linked to T2DM risk (adjusted OR = 2.61, 95% CI: 1.35–5.24, *P* = 0.006), whereas rs361525 showed no such association. Carriers of rs1800629 had higher mean TNF-α levels (*P* < 0.05), with A allele carriers among T2DM patients showing the greatest elevation. Our findings indicate that the rs1800629 and rs361525 variants may contribute meaningfully to MetS susceptibility, and that rs1800629, in particular, shows a notable association with T2DM risk, underscoring its potential relevance in personalized metabolic risk assessment.

## Introduction

Metabolic syndrome (MetS) represents an interconnected set of metabolic disturbances—ranging from adiposity-related changes to impaired glucose and lipid regulation—that collectively elevate the risk of downstream cardiometabolic diseases. The coexistence of these abnormalities substantially increases the risk of developing type 2 diabetes mellitus (T2DM) and cardiovascular disease (CVD), which together represent major contributors to global morbidity and mortality [1–3]. Rather than a single disease entity, MetS reflects systemic metabolic dysregulation arising from interactions between genetic susceptibility, chronic inflammation, and adverse lifestyle factors.

The global prevalence of metabolic syndrome (MetS) has increased substantially over the past two decades, driven by rising obesity rates, sedentary lifestyles, and population aging. Epidemiological analyses show a continual rise in MetS across diverse regions, including Asia, where demographic and lifestyle shifts have accelerated its emergence [4,5]. Current epidemiological data show that approximately 20%–40% of middle-aged and older adults in several Asian countries meet the criteria for MetS [6,7]. These findings highlight the growing public health burden of MetS in Asia and underscore the need for effective prevention and management strategies.

Growing evidence highlights the contribution of persistent, low-level inflammatory activity to the metabolic disturbances that characterize MetS. Among key proinflammatory mediators, tumor necrosis factor alpha (TNF-α) plays a pivotal role in linking adiposity to metabolic dysfunction. TNF-α is primarily secreted by macrophages, T lymphocytes, and natural killer cells and is also produced by adipose tissue under conditions of obesity. It disrupts insulin signaling pathways, enhances lipolysis, and promotes ectopic lipid accumulation, thereby contributing to systemic insulin resistance [8,9]. In addition, TNF-α has been implicated in pancreatic β-cell dysfunction and apoptosis, particularly in the presence of other inflammatory cytokines such as interleukin-1β and interferon-γ [10,11]. Elevated circulating TNF-α levels are consistently observed in individuals with obesity, MetS, and T2DM, and both experimental and clinical studies suggest that TNF-α inhibition can improve insulin sensitivity and metabolic outcomes [10–14].

The TNF-α gene, located in the class III region of the major histocompatibility complex on chromosome 6 [15], contains several promoter polymorphisms, including rs1800629 (−308 G > A) and rs361525 (−238 G > A), which may influence TNF-α expression and susceptibility to inflammatory and metabolic disorders, such as T2DM [16], MetS [17], systemic lupus erythematosus [15], chronic cervical spondylitis [18], and sepsis [19,20].

The rs1800629 A allele has been linked to increased TNF-α transcription and has been associated with obesity, insulin resistance, and T2DM in populations from Europe [21,22], China [23,24], and Egypt [25]. However, findings have been inconsistent, particularly among individuals of European ancestry [26–28].

In contrast, the functional relevance of rs361525 remains uncertain. Its effect on TNF-α expression has not been well established [29], and reported associations with MetS and T2DM have been inconsistent across different populations [30–33].

Given these uncertainties and the potential population-specific nature of these associations, this study aimed to investigate the relationship between TNF-α rs1800629 and rs361525 polymorphisms and MetS, T2DM, and related cardiometabolic risk factors in a Thai population. Additionally, serum TNF-α concentrations were assessed to elucidate the potential biological relevance of these variants.

## Materials and methods

### Study design and participants

This unmatched case–control study study included 850 Thai patients aged 25 years or older, drawn from the MetS–T2DM cohort at the HPH Princess Maha Chakri Sirindhorn Medical Center, Srinakharinwirot University. All participants were outpatients who visited the medical center between January 31, 2013, and January 30, 2014.

Metabolic syndrome (MetS) was diagnosed based on the 2009 Joint Interim Statement criteria proposed by the International Diabetes Federation (IDF) and related organizations. Participants were classified as having MetS if they met at least three of the following five criteria: (1) central obesity (waist circumference ≥90 cm in men or ≥80 cm in women, based on Asian-specific cut-offs), (2) elevated triglycerides (≥150 mg/dL or treatment for hypertriglyceridemia), (3) reduced HDL-C (<40 mg/dL in men, < 50 mg/dL in women), (4) elevated blood pressure (systolic ≥130 mmHg or diastolic ≥85 mmHg, or use of antihypertensive medication), (5) elevated fasting plasma glucose (≥100 mg/dL or diagnosis of type 2 diabetes mellitus). Body mass index (BMI) was included in data collection but not incorporated into the diagnostic criteria for central obesity. In accordance with WHO Asia-Pacific guidelines, a BMI ≥ 23 kg/m² was used as the threshold for overweight in Asian populations [34].

Individuals were excluded if they had any of the following: (1) underlying chronic inflammatory diseases (e.g., rheumatoid arthritis, systemic lupus erythematosus, inflammatory bowel disease); (2) active infections; (3) known malignancies; (4) chronic kidney disease stage 4 or higher; (5) liver cirrhosis; or (6) pregnancy. Patients receiving medications known to affect metabolic or inflammatory parameters were also excluded, including systemic corticosteroids, hormone therapies (e.g., hormone replacement therapy, oral contraceptives), insulin, thiazolidinediones, GLP-1 receptor agonists, DPP-4 inhibitors, and fibrates (e.g., fenofibrate). In particular, fibrate therapy was excluded due to its known effects on triglyceride levels and TNF-α concentrations, which were key variables in our analysis.

Of the 850 individuals initially screened, 808 met the initial eligibility criteria. Subsequently, 43 patients were excluded due to current fibrate use, leaving 765 participants for analysis. These comprised 344 MetS cases and 421 controls. The control group consisted of individuals without MetS who underwent the same assessments and met the same exclusion criteria.

All participants provided written informed consent before enrollment. This study was approved by the Faculty of Medicine, Srinakharinwirot University, Bangkok, Thailand (SWUEC-EX12/2556) and was conducted in accordance with the principles of the Declaration of Helsinki.

### Anthropometric and biochemical measurements

Anthropometric assessments included BMI (kg/m^2^), waist circumference, total body fat, and visceral fat. Waist circumference (WC) was measured with a flexible tape at the midpoint between the lower rib margin and the upper pelvic border. Total body fat and visceral fat were determined using bioelectrical impedance analysis with a body fat analyzer (Omron BF511; Omron Healthcare [UK] Ltd, Milton Keynes, UK).

Blood pressure, including systolic and diastolic values, was measured using an automated Udex-Twin device (Intertech Medical Group, Bangkok, Thailand). Participants were seated and allowed to rest for 10 minutes before measurement, following standard blood pressure protocols.

Venous blood samples were collected after a 12-hour fast. Fasting glucose, insulin, HbA1c, lipid profile, uric acid, high-sensitivity C-reactive protein, and microalbuminuria were measured with an Abbott Architect ci8200 Chemistry Analyzer (Abbott Laboratories, Green Oaks, IL, USA). Insulin resistance was calculated using the HOMA-IR. Serum TNF-α concentrations were determined with a human enzyme-linked immunosorbent assay kit (R&D Systems, Minneapolis, MN, USA) according to the manufacturer’s instructions. The Framingham risk score (FRS) was calculated based on age, sex, total cholesterol, HDL-C levels, history of hypertension treatment, systolic blood pressure, and smoking status.

### Genotyping

Genotyping was performed on 765 patients, focusing on two common variants in the promoter region of the TNF-α gene: rs1800629 (G/A) and rs361525 (G/A). Genomic DNA was extracted from peripheral blood leukocytes using the FlexiGene DNA Kit (Qiagen, Hilden, Germany) according to the manufacturer’s protocol. The extracted DNA was stored at −20 °C until further analysis. Both single nucleotide polymorphisms were genotyped by direct sequencing.

Sequencing reactions used BigDye Terminator v3.1 chemistry (Applied Biosystems, Foster City, CA, USA), and products were analyzed on an ABI 3730 Genetic Analyzer (Applied Biosystems). Sequence trace data were processed with the Staden Package (http://staden.sourceforge.net/) and independently verified by a second evaluator. Details of the primers and polymerase chain reaction protocols are provided in S1 Table.

### Statistical analysis

Baseline characteristics were summarized according to variable type. Categorical variables were reported as counts and percentages, and group comparisons were performed using the chi-square test. Continuous variables were expressed as medians with 95% confidence intervals (CIs), as their distributions were assessed to be non-normal using the Shapiro-Wilk test. Comparisons between groups were conducted using the Mann–Whitney U test. Groups were defined based on the presence or absence of metabolic syndrome (MetS), its individual components (central obesity, dyslipidemia, hypertension, and hyperglycemia), as well as other cardiometabolic risk factors, including T2DM.

Multiple logistic regression analyses were performed to assess the associations between TNF-α variants and disease status (MetS and T2DM), adjusting for sex, age, and BMI. Crude and adjusted odds ratios (ORs and aORs) with 95% CIs were calculated.

All statistical analyses were performed using R (version 4.1.2; R Foundation for Statistical Computing, Vienna, Austria). To compare serum TNF-α concentrations, mean values and standard errors of the mean were calculated, and group differences were assessed using the Mann‒Whitney *U* test in GraphPad Prism (version 8.4.3; GraphPad Software Inc, Boston, MA, USA). Statistical significance was set at *P* < 0.05. Hardy‒Weinberg equilibrium was tested using a fast exact test [35]. Additionally, haplotype analysis of the TNF-α variants (rs1800629 and rs361525) was performed using the haplo.stats package in R [36], which estimates haplotype frequencies and examines their associations through logistic regression analysis.

## Results

### Participant characteristics

We included a total of 765 participants in our study, comprising 421 individuals without MetS and 344 with MetS. The baseline characteristics by MetS status are presented in Table 1.

**Table 1:** Demographic and biochemical characteristics of subjects stratified by Metabolic syndrome (MetS) status.

Parameters	Non-MetS *n* = 421	MetS * n = 344	*P* value*
Sex (F/M)^†^	293/128	211/133	***0.02***
Age (y)	43 (41–47)	60 (58–61)	***<0.001***
BMI (kg/m^2^)	22.76 (22.41-23.11)	27.73 (27.34-28.3)	***<0.001***
WC (cm)	80 (79-82)	94 (93-95)	***<0.001***
TBF (%)	28.9 (28.1-29.3)	34.45 (33.5-35.2)	***<0.001***
VF (%)	6 (6–7)	14 (13–14)	***<0.001***
SBP (mmHg)	115 (113-117)	132 (130-133)	***<0.001***
DBP (mmHg)	70 (68 –72)	77 (74-78)	***<0.001***
PWV left (cm/s)	1333.5 (1287.5-1367)	1649 (1591-1685)	***<0.001***
PWV right (cm/s)	1306.5 (1274-1348)	1639 (1588-1683)	***<0.001***
FBG (mg/dL)	94 (92-94)	119 (116-123)	***<0.001***
HbA1c (%)	5.5 (5.3-5.5)	6.1 (6-6.2)	***<0.001***
Fasting insulin (mU/L)	12.1 (11.4-12.7)	15.9 (14.9-16.9)	***<0.001***
TC (mg/dL)	194 (190.5-199)	172 (168-175.5)	***<0.001***
TG (mg/dL)	95 (89-100.5)	139.5 (129.5-148)	***<0.001***
LDL-C (mg/dL)	124 (119-128)	103 (99-110)	***<0.001***
HDL-C (mg/dL)	60 (57.5-62)	49 (47–50)	***<0.001***
Uric acid (mg/dL)	4.97 (4.81-5.18)	5.92 (5.72-6.17)	***<0.001***
MAU (mg/g Cr)	5.98 (5.47-6.77)	11.79 (10.17-13.86)	***<0.001***
hs-CRP (mg/L)	1.24 (1-1.42)	1.86 (1.43-2.14)	***<0.001***
Framingham risk score (%)	0.1 (0.1-0.1)	3 (3–4)	***<0.001***
History of HT^†^	102 (24.2%)	273 (79.4%)	***<0.001***
History of DLP^†^	49 (11.6%)	160 (45.5%)	***<0.001***
History of T2DM^†^	118 (28.0%)	309 (89.83%)	***<0.001***

**Abbreviations:** BMI, body mass index; DBP, diastolic blood pressure; DLP, dyslipidemia; FBG, fasting blood glucose; HbA1c, glycated hemoglobin; HDL-C, high-density lipoprotein cholesterol; hs-CRP, high-sensitivity C-reactive protein; HT, hypertension; LDL-C, low-density lipoprotein cholesterol; MAU, microalbuminuria; MetS, metabolic syndrome; PWV, pulse wave velocity; SBP, systolic blood pressure; T2DM, type 2 diabetes mellitus; TBF, total body fat; TC, total cholesterol; TG; triglycerides, VF, visceral fat; WC, waist circumference.

Metabolic syndrome (MetS) was defined according to the 2009 International Diabetes Federation criteria. Hypertension was defined as systolic blood pressure ≥130 mmHg or diastolic blood pressure ≥85 mmHg. Dyslipidemia was defined as elevated triglycerides (≥150 mg/dL), reduced HDL cholesterol (<40 mg/dL in men or <50 mg/dL in women). Type 2 diabetes mellitus (T2DM) was diagnosed according to the American Diabetes Association criteria.

The data are presented as the median and 95% confidence interval of the median. * *P* values were evaluated by the Mann–Whitney U test, except for sex (expressed as F/M ratio and other categorical variables. † *P* values were evaluated by the chi-square test. Significant *P* values are presented in bold and italics.

We observed that the prevalence of T2DM was significantly higher in the MetS group (89.8%) compared to the non-MetS group (28.0%, *P* < 0.001). Participants classified with MetS displayed an older age profile and showed elevations across several cardiometabolic indicators, including blood pressure, overall adiposity, and central fat accumulation.

Markers of glucose dysregulation were notably accentuated in the MetS subgroup, as evidenced by significantly higher fasting insulin concentrations and elevated homeostatic model assessment of insulin resistance (HOMA-IR) values. In addition, they had elevated levels of fasting blood glucose (FBG) and glycated hemoglobin (HbA1c).

Interestingly, despite a higher prevalence of metabolic syndrome, the MetS group showed lower TC and LDL-C levels than the non-MetS group. This pattern, noted in clinical populations, may stem from lipid-lowering therapy in those with metabolic issues. Thus, these results warrant cautious interpretation. Markers of metabolic dysfunction, including uric acid, microalbuminuria, and high-sensitivity C-reactive protein (hs-CRP), were also significantly increased (*P* < 0.05 for all).

Notably, we found that the proportion of female participants was significantly higher in the non-MetS group.

### Associations between TNF-α genetic variants and clinical, biochemical parameters

Baseline clinical and biochemical characteristics stratified by TNF-α genotypes (rs1800629 and rs361525) are presented in Table 2. Among rs1800629 genotypes, participants harboring the rs1800629 A allele (G/A + A/A) demonstrated significantly higher age, fasting glucose, and bilateral PWV compared to G/G homozygotes. Furthermore, these individuals showed consistent increases in estimated cardiovascular risk, as reflected by higher Framingham risk scores (P < 0.05). Genotypic groups showed no significant differences across a wide range of metabolic and inflammatory parameters. This lack of phenotypic impact extended to blood pressure, obesity indices (BMI and visceral fat), fasting insulin, HOMA-IR, and full lipid profiles. Additionally, markers such as HbA1c, uric acid, microalbuminuria, and hs-CRP remained comparable between groups.

**Table 2:** Demographic and biochemical characteristics of patients stratified by tumor necrosis factor alpha variants rs1800629 and rs361525.

Parameters	rs1800629			rs361525		
	Major allele homozygote (*n* = 665) GG	Minor allele carriers (*n* = 100) GA + AA	*P* value*	Major allele homozygote (*n* = 728) GG	Minor allele carriers (*n* = 37) GA	*P* value*
Sex (F/M) ^†^	444/221	60/40	0.223	479/249	25/12	0.965
Age (y)	53 (52–55)	58 (53.5-61)	***0.029***	54 (52–55)	54 (44–60)	0.919
BMI (kg/m^2^)	24.69 (24.24-25.08)	25.22 (24.24-26.08)	0.616	24.71 (24.27-25.08)	25.78 (24.09-27.92)	0.366
WC (cm)	86 (84-87)	86 (84.5-89.5)	0.190	86 (85-87)	89 (85-94)	0.250
TBF (%)	30.8 (30.2-31.8)	31.6 (29.7-33.9)	0.594	30.8 (30.2-31.8)	30.9 (28.7-33.3)	0.925
VF (%)	10 (9–10)	10 (9–11)	0.332	10 (9–10)	13 (9–14)	0.230
SBP (mmHg)	123 (121-125)	124.5 (121.5-130)	0.239	124 (122-125)	118 (108-127)	0.211
DBP (mmHg)	73 (72-74)	74.5 (70.5-77)	0.907	73 (72-74)	73 (69-79)	0.770
PWV left (cm/s)	1464 (1434-1498)	1560 (1521-1642)	***0.004***	1481 (1450-1509)	1562 (1404-1664)	0.273
PWV right (cm/s)	1465 (1435-1498)	1562 (1490-1654)	***0.007***	1475.5 (1444-1506.5)	1561 (1455-1711)	0.109
FBG (mg/dL)	103 (100-105)	112 (107.5-115)	***0.003***	104 (102-106)	109 (100-116)	0.482
HbA1c (%)	5.8 (5.8-5.9)	5.9 (5.8-6.1)	0.082	5.9 (5.8-5.9)	5.8 (5.65-6)	0.884
Fasting insulin (mU/L)	13.3 (12.8-13.85)	14.05 (12.4-15.3)	0.496	13.4 (12.9-14)	12.75 (10.9-16.55)	0.847
TC (mg/dL)	186 (182-189)	186.5 (166.5-198)	0.326	186 (182-189)	188 (176-206)	0.635
TG (mg/dL)	110 (105-117)	123 (100-130)	0.306	111 (106-118)	110 (89-139)	0.871
LDL-C (mg/dL)	117 (114-120)	110.5 (99.5-123)	0.328	116 (112.5-119)	116 (100-128)	0.948
HDL-C (mg/dL)	55 (53–56)	52 (49.5-59)	0.359	54 (53–56)	55 (45-64)	0.859
Uric acid (mg/dL)	5.35 (2.24-5.56)	5.69 (5.3-6.04)	0.272	5.38 (5.28-5.56)	5.56 (4.99-5.97)	0.779
MAU (mg/g Cr)	8.34 (7.49-9.35)	10.12 (6.61-14.56)	0.376	8.39 (7.62-9.47)	10.91 (5.17-19.26)	0.560
hs-CRP (mg/L)	1.42 (1.3-1.88)	1.32 (1.08-1.95)	0.580	1.42 (1.30-1.87)	1.38 (0.84-1.96)	0.489
Framingham risk score (%)	1 (1–2)	2 (1–4)	***0.038***	1 (1–2)	2 (0.1-3)	0.438

**Abbreviations:** BMI, body mass index; DBP, diastolic blood pressure; FBG, fasting blood glucose; HbA1c, glycated hemoglobin; HDL-C, high-density lipoprotein cholesterol; hs-CRP, high-sensitivity C-reactive protein; LDL-C, low-density lipoprotein cholesterol; MAU, microalbuminuria; PWV, pulse wave velocity; SBP, systolic blood pressure; TBF, total body fat; TC, total cholesterol; TG; triglycerides, VF, visceral fat; WC, waist circumference.

The data are presented as the median and 95% confidence interval of the median

* *P* values were evaluated by the Mann–Whitney *U* test, except for sex (expressed asF/M ratio)

† *P* values were evaluated by the chi-square test.

Significant *P* values are presented in bold and italics.

Similarly, carriers of the rs361525 minor allele (G/A) did not differ significantly from G/G homozygotes across any clinical or biochemical parameters.

### Associations between TNF-α variants and cardiometabolic risk factors

Logistic regression analyses (Table 3) demonstrated that the rs1800629 variant was significantly associated with increased odds of insulin resistance, as measured by HOMA-IR (OR = 1.78, P = 0.021), and hypertension (OR = 2.26, P = 0.005). These associations remained statistically significant after adjustment for age, sex, and BMI. In contrast, the rs361525 variant showed no significant association with insulin resistance, hypertension, or any obesity-related traits (Table 3).

**Table 3:** Associations between tumor necrosis factor alpha variants rs1800629 and rs361525 and clinical cardiometabolic risk factors.

Cardiometabolic risks	rs1800629				rs361525			
	Minor allele carriers versus major allele homozygous				Minor allele carriers versus major allele homozygous			
	Crude		Adjusted *		Crude		Adjusted *	
	OR (95% CI)	*P* value ^‡^	OR (95% CI)	*P* value ^‡^	OR (95% CI)	*P* value ^‡^	OR (95% CI)	*P* value ^‡^
MetS *	2.22 (1.45-3.44)	***<0.001***	3.13 (1.78-5.60)	***<0.001***	2.08 (1.07-4.21)	***0.035***	2.84 (1.17-7.31)	***0.025***
T2DM *	2.30 (1.45-3.75)	***<0.001***	2.61 (1.35-5.24)	***0.006***	1.15 (0.57-2.39)	0.709	1.23 (0.46-3.46)	0.688
HOMA-IR *	1.69 (1.09-2.63)	***0.019***	1.78 (1.10-2.91)	***0.021***	1.03 (0.53-2.03)	0.921	0.91 (0.42-1.94)	0.799
High TG *	1.24 (0.79-1.92)	0.346	1.14 (0.71-1.80)	0.587	1.13 (0.54-2.24)	0.738	1.09 (0.51-2.23)	0.821
Low HDL-C *	1.17 (0.72-1.84)	0.513	1.21 (0.73-1.96)	0.451	0.79 (0.33-1.69)	0.568	0.69 (0.28-1.54)	0.392
Hypertension *	2.13 (1.38-3.33)	***<0.001***	2.26 (1.30-4.02)	***0.005***	1.24 (0.64-2.43)	0.531	1.23 (0.54-2.85)	0.628
High PWV right *	1.70 (1.08-2.73)	***0.024***	1.35 (0.73-2.57)	0.349	1.83 (0.90-4.03)	0.110	2.36 (0.90-6.75)	0.092
High PWV left *	2.12 (1.33-3.49)	***0.020***	1.97 (0.88-4.42)	0.101	1.58 (0.79-3.38)	0.214	1.80 (0.73-4.74)	0.216
High BMI ^†^	1.23 (0.80-1.87)	0.343	1.11 (0.72-1.71)	0.650	1.47 (0.76-2.91)	0.255	1.52 (0.77-3.04)	0.229
High WC ^†^	1.11 (0.73-1.71)	0.617	1.12 (0.70-1.78)	0.642	1.50 (0.76-3.07)	0.252	1.57 (0.76-3.39)	0.236
High VF ^†^	1.10 (0.72-1.68)	0.666	0.81 (0.50-1.32)	0.399	1.43 (0.74-2.86)	0.293	1.62 (0.77-3.50)	0.212
High uric acid *	1.30 (0.85-1.99)	0.22	1.15 (0.70-1.88)	0.588	1.21 (0.61-2.37)	0.582	1.26 (0.57-2.76)	0.566
MAU *	1.35 (0.77-2.28)	0.277	1.31 (0.74-2.25)	0.338	1.71 (0.73-3.70)	0.192	1.92 (0.80-4.28)	0.125
hs-CRP *	0.85 (0.34-1.86)	0.702	0.87 (0.34-1.92)	0.739	0.49 (0.08-1.71)	0.340	0.47 (0.07-1.67)	0.319
FRS *	1.46 (0.84-2.43)	0.160	1.17 (0.51-2.60)	0.707	0.46 (0.11-1.30)	0.202	0.49 (0.08-2.29)	0.395

**Abbreviations:** BMI, body mass index; FRS, Framingham risk score; HDL-C, high-density lipoprotein cholesterol; HOMA-IR, homeostatic model assessment of insulin resistance; hs-CRP, high-sensitivity C-reactive protein; MAU, microalbuminuria; MetS, metabolic syndrome; PWV, pulse wave velocity; T2DM, type 2 diabetes mellitus; TBF, total body fat; TC, total cholesterol; TG; triglycerides, VF, visceral fat; WC, waist circumference

Metabolic syndrome (MetS) was defined according to the 2009 International Diabetes Federation criteria. Type 2 diabetes mellitus (T2DM) was diagnosed according to the American Diabetes Association criteria. High triglycerides (TG) were defined as levels ≥ 150 mg/dL. The threshold for low HDL-C was set at < 40 mg/dL in men and < 50 mg/dL in women. High PWV was defined > 1400 cm/s. Hypertension was defined as systolic blood pressure ≥ 140 mmHg or diastolic blood pressure ≥ 90 mmHg. A high BMI was defined as a BMI ≥ 23 kg/m^2^. High WC was defined as >90 cm for men and >80 cm for women. Insulin resistance was set at the 50th percentile of HOMA-IR in this study (HOMA-IR ≥ 3.50). hs-CRP levels above 3.0 mg/L were classified as elevated. High uric acid ≥ 7.2 mg/dL. Microalbuminuria (MAU) was defined ≥ 30 mcg/mg creatinine. High visceral fat rating ≥ 10. High pulse wave velocity ≥ 1400 cm/s. The Framingham risk score (FRS) was ≥ 10%.

* Adjusted by age, sex, and body mass index. † Adjusted by age and sex.

‡ Significant *P* values are presented in bold and italics.

### Associations between TNF-α genetic variants and MetS

Participants were classified based on MetS status, and genotype frequencies for rs1800629 and rs361525 are presented in S2 Table. The rs1800629 A allele was significantly more frequent among individuals with MetS (9.5%) compared to those without MetS (4.8%). Logistic regression analysis, adjusted for age, sex, and BMI, confirmed a significant association between rs1800629 and increased odds of MetS (adjusted OR = 3.13, *P* < 0.001; Table 3), with consistent results observed under the dominant, allelic, and log-additive genetic models (Table 4). Notably, this association appeared to be independent of general adiposity, as no significant differences in genotype distribution were observed across MetS strata (S3 Table).

**Table 4:** Associations between tumor necrosis factor alpha variants and metabolic syndrome status.

Variants	Frequency (%)		Non-MetS vs MetS *			
	Non-MetS *n* = 421	MetS * n = 344	OR (95% CI)	*P* value ^†^	OR ** (95% CI)	*P* value ^†^
rs1800629 (G>A)						
Genotype						
Dominant						
G/G	383	282	1.00	–	1.00	–
G/A+A/A	38	62	2.22 (1.45-3.44)	***<0.001***	3.13 (1.78-5.16)	***<0.001***
Recessive						
G/A+G/G	419	341	1.00	–	1.00	–
A/A	2	3	1.84 (0.3-14.05)	0.50		
Allele						
G	802	623	1.00	–	1.00	–
A	40	65	2.09 (1.40-3.17)	***<0.001***	2.85 (1.68-4.90)	***<0.001***
Log additive						
	–	–	2.99 (1.65-5.52)	***<0.001***	4.96 (2.25-11.26)	***<0.001***
rs361525 (G>A)						
Genotype						
Dominant						
G/G	407	321	1.00	–	1.00	–
G/A	14	23	2.08 (1.07-4.21)	***0.035***	2.84 (1.17-7.31)	***0.025***
Allele						
G	828	665	1.00	–	1.00	–
A	14	23	2.05 (1.06-4.10)	***0.037***	2.76 (1.15-6.99)	***0.027***
Log additive						
	–	–	2.88 (1.10-7.95)	***0.035***	4.51 (1.25-17.62)	***0.025***

**Abbreviations:** 95% CI, 95% confidence interval; MetS, metabolic syndrome; OR, odds ratio; T2DM, type 2 diabetes mellitus

Metabolic syndrome (MetS) was defined according to the 2009 International Diabetes Federation criteria.

** Adjusted by age, sex, and body mass index.

† Significant *P* values are presented in bold and italics.

For rs361525, the minor A allele was more prevalent among individuals with MetS (3.3%) compared to controls (1.7%), a difference that reached statistical significance (P < 0.05). After adjustment for age, sex, and BMI, logistic regression analysis revealed a significant association between rs361525 and increased odds of MetS (adjusted OR = 2.84, P = 0.025; Table 3), with consistent findings observed under the dominant, allelic, and log-additive genetic models (Table 4).

### Associations between TNF-α genetic variants and T2DM

Participants were categorized based on the presence or absence of T2DM, with genotype frequencies for rs1800629 and rs361525 presented in S3 Table. The rs1800629 A allele was significantly more prevalent among individuals with T2DM (8.9%) compared to those without T2DM (4.3%). Logistic regression analysis, adjusted for age, sex, and BMI, confirmed a significant association between rs1800629 and increased odds of T2DM (adjusted OR = 2.61, *P* = 0.006; Table 3), with consistent findings observed under the dominant, allelic, and log-additive genetic models (Table 5). Similar to the findings for MetS, this association appeared to be independent of obesity, as no significant differences in genotype distribution were observed across T2DM strata (S4 Table).

**Table 5:** Associations between tumor necrosis factor alpha variants and type 2 diabetes mellitus status.

Variants	Frequency (%)		Non-T2DM vs T2DM *			
	Non-T2DM *n* = 338	T2DM *n* = 427	OR (95% CI)	*P* value ^†^	OR ** (95% CI)	*P* value ^†^
rs1800629 (G > A)						
Genotype						
Dominant						
G/G	311	354	1.00	–	1.00	–
G/A + A/A	27	73	2.38 (1.51-3.85)	***<0.001***	2.80 (1.51-5.39)	***0.001***
Recessive						
G/A + G/G	336	424	1.00	–	1.00	–
A/A	2	3	1.19 (0.20-9.06)	0.85	1.39 (0.11-25.42)	0.81
Allele						
G	647	778	1.00	–	1.00	–
A	29	76	2.18 (1.42-3.43)	***<0.001***	2.54 (1.42-4.69)	***0.002***
Log additive						
	–	–	3.22 (1.71-6.30)	***<0.001***	4.08 (1.72-10.15)	***0.002***
rs361525 (G > A)						
Genotype						
Dominant						
G/G	321	407	1.00	–	1.00	–
G/A	17	20	0.93 (0.48-1.82)	0.83	0.72 (0.31-1.73)	0.45
Allele						
G	659	834	1.00	–	1.00	–
A	17	20	0.93 (0.48-1.81)	0.83	0.73 (0.32-1.72)	0.46
Log additive						
	–	–	0.90 (0.48-1.82)		0.62 (0.19-2.20)	0.45

**Abbreviations:** 95% CI, 95% confidence interval; OR, odds ratio; T2DM, type 2 diabetes mellitus.

* Type 2 diabetes mellitus (T2DM) was diagnosed according to the American Diabetes Association criteria.

** Adjusted by age, sex, and body mass index.

† Significant *P* values are presented in bold and italics.

In contrast, rs361525 showed no significant association with T2DM under any genetic model (Table 5).

### Haplotype analysis of TNF-α variants (rs1800629 and rs361525) and disease status (MetS and T2DM)

Haplotype analysis of rs1800629 and rs361525, adjusted for age, sex, and body mass index, revealed that the A/G haplotype was significantly associated with an increased risk of both MetS (adjusted OR = 1.92, P = 0.006) and T2DM (adjusted OR = 2.01, P = 0.015) (Table 6). The A/A haplotype was not detected in this cohort, likely due to its low allele frequency.

**Table 6:** Associations between tumor necrosis factor alpha haplotype patterns and metabolic syndrome and type 2 diabetes mellitus.

Haplotype (rs1800629–rs361525)	MetS * (frequency)	Non-MetS (frequency)	Crude		Adjusted ^‡^	
			OR (95% CI)	*P* value **	OR (95% CI)	*P* value **
G/G	0.8773	0.9359	1.00	–	1.00	–
A/G	0.0893	0.0475	1.98 (1.31–3.00)	***0.001***	1.92 (1.21–3.04)	***0.006***
G/A	0.0282	0.0166	1.87 (0.92–3.8)	0.08	1.93 (0.95–3.93)	0.07
Haplotype (rs1800629–rs361525)	T2DM ^†^ (frequency)	No-T2DM (frequency)	OR (95% CI)	*P* value **	OR (95% CI)	*P* value **
G/G	0.8921	0.932	1.00	–	1.00	–
A/G	0.0845	0.0429	2.02 (1.20–3.16)	***0.002***	2.01 (1.44–3.53)	***0.015***
G/A	0.0189	0.022	0.80 (0.40–1.62)	0.54	0.84 (0.36–1.95)	0.68

**Abbreviations:** 95% CI, 95% confidence interval; MetS, metabolic syndrome; OR, odds ratio; T2DM, type 2 diabetes mellitus.

* Metabolic syndrome (MetS) was defined according to the 2009 International Diabetes Federation criteria.

† Type 2 diabetes mellitus (T2DM) was diagnosed according to the American Diabetes Association criteria.

‡ Adjusted by age, sex, and body mass index.

** Significant *P* values are presented in bold italic.

### Association between TNF-α variants and circulating TNF-α levels

**Fig 1:**
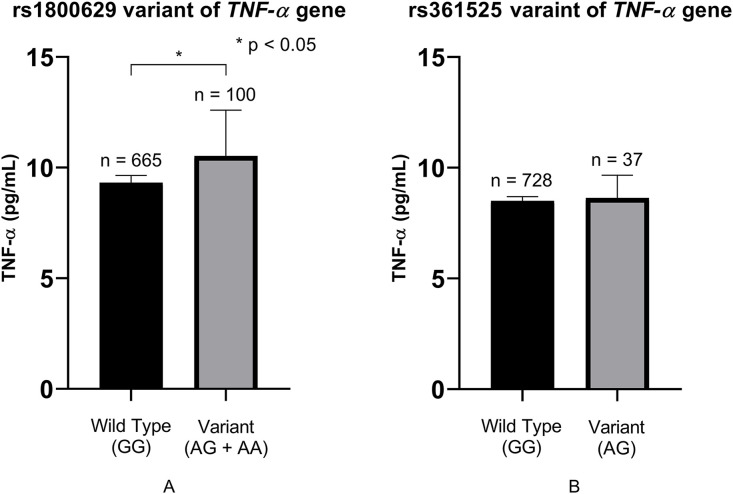
Comparative analysis of serum TNF-α concentrations across different genetic variants. **(A)** rs1800629 variant of the *TNF-α* gene. Serum TNF-α levels (pg/mL) are significantly higher in individuals carrying the variant alleles (AG + AA, n = 100) compared to those with the Wild Type (GG, n = 665) genotype. The asterisk (*) indicates a statistically significant difference with a *P*value < 0.05. B) rs361525 variant of the *TNF-α* gene: No significant difference in serum TNF-α concentrations was observed between the Wild type (GG, n = 728) and the Variant (AG, n = 37) groups.

To explore the functional relevance of TNF-α variants, we compared serum TNF-α levels across genotypes. Individuals carrying the rs1800629 A allele exhibited significantly higher TNF-α concentrations compared to G/G homozygotes (10.52 pg/mL vs. 9.32 pg/mL, P < 0.05; Fig 1A). In contrast, no significant differences in TNF-α levels were observed across rs361525 genotypes (Fig 1B).

## Discussion

Prior investigations from various global cohorts have linked TNF-α polymorphisms to a range of metabolic traits [21,22,24,25,29–32], however, these associations had not yet been fully explored in the Thai population. To address this knowledge gap, the present study investigated the relationship between the *TNF-α* variants rs1800629 and rs361525 and their impact on MetS, T2DM, and related cardiometabolic risk factors within a Thai cohort.

In our analysis, the rs1800629 variant displayed measurable links to several metabolic traits, including indicators of cardiometabolic strain and glycemic dysregulation. However, it was not associated with high triglycerides, low HDL-C, high BMI, large waist circumference, high visceral fat, elevated uric acid, microalbuminuria, or high-sensitivity C-reactiveprotein. After adjusting for age and sex, MetS, T2DM, insulin resistance, hypertension, and high pulse wave velocity remained significantly associated with rs1800629 (Table 3).

Previous research has extensively investigated the association of rs1800629 with T2DM in White and other populations [21,22,24,25]. However, its relevance to the Thai population remained unknown. The observed associations are in line with patterns documented in earlier international studies, suggesting that the metabolic influence of rs1800629 extends across diverse populations. Our findings specifically corroborate previous research in White and other ethnic groups, further validating the association between the rs1800629 variant and both MetS[24,33,37,38] and T2DM [39–43].

In this study, we identified a significant association between rs1800629 and increased risk of MetS across dominant, allelic, and log-additive genetic models (Table 4). When examining different inheritance model frameworks, rs1800629 consistently demonstrated elevated effect estimates, reinforcing its potential biological contribution to metabolic risk. In our dominant model, the rs1800629 variant was associated with an over threefold increase in risk (OR = 3.13; 95% CI: 1.78–5.16, *P* < 0.0001), a finding consistent with previous research reporting an OR of 2.75 [25]. Similarly, the allelic model showed a strong significant association (OR = 2.85; 95% CI: 1.68–4.90, *P* = 0.0001), aligning with earlier reported values (OR = 5.39), although the magnitude of effect differed [25]. Notably, the proportion of individuals with T2DM was not reported in that earlier study, a factor that may limit direct comparability between these effect sizes.

In contrast, Zafar et al. [44] studying an Indian population, found no significant association between rs1800629 and MetS. Their study also did not report T2DM prevalence, and the A allele frequency was similar between MetS cases and healthy controls. No significant associations were observed under any genetic model, suggesting potential population-specific effects or differences in phenotype definitions, study design, or underlying genetic architecture.

We also observed a significant association between rs1800629 and increased risk of T2DM across multiple genetic models (Table 5). In the dominant model, the OR was 2.38 (95% CI: 1.51–3.85, P = 0.0003), which is comparable to a previous study reporting an OR of 1.63 [45], where 50% of participants were diagnosed with T2DM. Under the allelic model, the OR was 2.18 (95% CI: 1.42–3.43, P = 0.0005), aligning with earlier findings (OR = 1.56) [32]. The log-additive model yielded an OR of 4.08 (95% CI: 1.72–10.15, P = 0.002), indicating a stronger effect in our cohort compared to the previously reported OR of 1.56 [45].

To investigate whether the association between rs1800629 and cardiometabolic outcomes is mediated by general obesity, we conducted subgroup analyses stratified by MetS and T2DM status. Notably, variation at rs1800629 did not appear to influence overall adiposity levels, as evidenced by the lack of significant association with BMI in either subgroup (S4 Table). These findings suggest that the impact of rs1800629 on MetS and T2DM risk is likely independent of general adiposity, potentially arising through metabolic or inflammatory pathways unrelated to obesity.

For rs361525, the metabolic profile was largely comparable across genotypes, indicating minimal observable impact on the phenotypes measured in this cohort. Specifically, no significant associations were observed between this variant and T2DM or any individual components of MetS, including lipid profiles (triglycerides and HDL-C), obesity indices (BMI, waist circumference, and visceral fat), and clinical markers such as uric acid, microalbuminuria, or systemic inflammation (hs-CRP). Furthermore, rs361525 did not appear to influence overall cardiovascular risk, as reflected by the Framingham risk scores (FRS) (Table 3). These findings are consistent with previous studies conducted in Indian populations, where rs361525 was similarly unassociated with metabolic parameters such as lipid profiles, obesity measures, and hs-CRP levels, 14.8% of participants in one study being diagnosed with T2DM [29].

We observed a significant association between the rs361525 variant and MetS, despite the absence of associations with individual MetS components such as waist circumference, triglycerides, HDL-C, or blood pressure (Tables 3 and 4). This finding suggests that rs361525 may contribute to MetS through cumulative or synergistic effects on subclinical metabolic abnormalities that, while individually nonsignificant, collectively fulfill the diagnostic criteria for MetS. Notably, the A allele of rs361525, located in a regulatory promoter region, has previously been associated with postprandial elevations in free fatty acids and insulin resistance in obese individuals with T2DM [46].

Moreover, gene–diet interactions have been reported, with the A allele influencing lipid metabolism in response to dietary fat quality [47]. Although dietary intake and methylation status were not assessed in our study, previous research has demonstrated that methylation of the TNF-α promoter region can modulate gene expression [48]. These findings suggest that rs361525 may influence susceptibility to MetS through complex gene–environment and epigenetic mechanisms, rather than through direct effects on individual MetS components.

Our haplotype analysis revealed that the A/G haplotype—comprising the rs1800629 A allele and the rs361525 G allele—was significantly associated with an increased risk of both MetS and T2DM, whereas the G/A haplotype showed no association with either condition (Table 6). While this pattern may suggest a potential interaction between the rs1800629 A allele and the rs361525 G allele, it is also plausible that the observed association is primarily driven by the rs1800629 variant, given the lack of a significant individual association between rs361525 and T2DM.

The absence of the A/A haplotype in our cohort precluded the assessment of potential additive or synergistic effects. This likely reflects the low population frequency of this haplotype and underscores the need for larger studies to elucidate gene–gene interactions and their contributions to cardiometabolic risk.

TNF-α is a key cytokine in the immune system, primarily produced by macrophages, T cells, and other immune cells. It regulates inflammation, cell survival, and apoptosis [9,49]. TNF-α impairs insulin signaling by phosphorylating insulin receptor substrates, which reduces glucose uptake in muscle and adipose tissues. This process contributes to insulin resistance, a hallmark of MetS and T2DM [50–52]. Additionally, elevated TNF-α levels are associated with poor glycemic control in T2DM, with a significant correlation observed between serum TNF-α levels and HbA1c [8,53].

The rs1800629 variant enhances gene transcription by creating a binding site for NF-κB, resulting in increased TNF-α production and heightened inflammation [54]. In contrast, the rs361525 variant has minimal influence on TNF-α expression, because its G allele does not alter transcription factor binding or promoter activity [55].

An *ex vivo* study reported that whole blood samples from carriers of the rs1800629 A allele produced higher levels of TNF-α following lipopolysaccharide stimulation compared to homozygous G allele carriers [56]. Furthermore, a clinical study showed an association between the rs1800629 A allele and elevated serum TNF-α in asthmatic patients [24]. To validate these observations, we measured serum TNF-α concentrations in carriers of the rs1800629 A allele (GA or AA genotypes) and found significantly elevated levels compared to non-carriers (Fig 1A). These results support the hypothesis that rs1800629 contributes to increased TNF-α expression and may underlie enhanced systemic inflammation. In contrast, a previous study reported no association between rs1800629 and TNF-α levels [57]. This discrepancy may be attributable to differences in study conditions, including the clinical characteristics of the cohorts. Notably, the proportion of participants with T2DM was 39.5% in that study, compared to a higher prevalence in our cohort (55.8%). Such differences in disease burden and population characteristics may influence genotype–phenotype associations and could partly explain the observed inconsistencies. These findings underscore the need for further studies in diverse populations to clarify the role of rs1800629 in TNF-α expression and metabolic risk.

Elevated TNF-α levels are strongly associated with both MetS and T2DM. Individuals with MetS exhibit significantly higher TNF-α concentrations compared to healthy controls [58]. Furthermore, individuals with both T2DM and obesity exhibit markedly elevated TNF-α levels compared to those with T2DM but without obesity, as well as individuals without diabetes and without obesity. In that study, 50.7% of participants were diagnosed with T2DM. Notably, increased TNF-α levels were positively correlated with HbA1c and insulin resistance, as measured by HOMA-IR [8].

Our findings support the role of TNF-α gene variants, along with additional genetic and environmental factors, in determining the risk of MetS and T2DM. These variants may serve as useful biomarkers and potential therapeutic targets for prevention, nutritional interventions, and treatment strategies. However, the study is limited by its lack of longitudinal follow-up and the absence of a replication cohort. Further research is needed to clarify the mechanisms by which rs1800629 and rs361525 increase disease susceptibility.

## Conclusions

Our findings demonstrate that variations at rs1800629 and rs361525 within the TNF-α gene are associated with an increased susceptibility to MetS among Thai adults. Notably, carriers of the rs1800629 A allele exhibited a significantly higher likelihood of developing T2DM, an effect that appeared independent of general adiposity. These observations suggest that TNF-α polymorphisms may serve as valuable genetic markers for refining metabolic risk assessment and implementing individualized preventive strategies in the Thai population. To further elucidate the underlying biological mechanisms, future investigations incorporating functional assays and longitudinal follow-up are warranted to confirm the clinical utility of these variants in predicting long-term cardiometabolic outcomes.

## Supporting information

S1 Table Primers and polymerase chain reaction protocols for tumor necrosis factor alpha single nucleotide polymorphisms rs1800629 and rs361525. (PDF)

S2 Table Tumor necrosis factor alpha variants, genotypes, minor allele frequency, and Hardy–Weinberg equilibrium test stratified by metabolic syndrome status. (PDF)

S3 Table Tumor necrosis factor alpha variants, genotypes, minor allele frequency, and Hardy–Weinberg equilibrium test stratified by type 2 diabetes mellitus status. (PDF)

S4 Table Logistic regression analysis of rs1800629 genotypes and their association with obesity, stratified by disease status (MetS and T2DM). (PDF)

## Abbreviation:

BMI: body mass index
HbA1c: glycated hemoglobin
HDL-C: high-density lipoprotein cholesterol
HOMA-IR: homeostatic model assessment of insulin resistance
MetS: metabolic syndrome
T2DM: type 2 diabetes mellitus
TNF-α: tumor necrosis factor alpha
