# Supplementary material for: Associations of tumor necrosis factor alpha genetic variants with metabolic syndrome and type 2 diabetes mellitus in a Thai population
Source: PLoS One. 2026 Apr 2;21(4):e0346147. doi: 10.1371/journal.pone.0346147 (PMC13046163; doi:10.1371/journal.pone.0346147)
Supplement: S1 Table — (PDF) [file pone.0346147.s001.pdf]

S1 Table. Primers and polymerase chain reaction protocols for tumor necrosis factor alpha single nucleotide polymorphisms rs1800629 and rs361525.

Gene	Target primer	Sequence (5'→3')	PCR protocol
TNF-α	rs1800629		
	Forward	AACACAGCTTTTCCCTCCAA	95 °C for 5 min; 35 cycles at 94 °C for 30 s, 57 °C for 40 s, and 72 °C for 45 s; followed by 72 °C for 10 min
	Reverse	TAGCTGGTCCTCTGCTGTCC	
	rs361525		
	Forward	AACACAGCTTTTCCCTCCAA	95 °C for 5 min; 35 cycles at 94 °C for 30 s, 57 °C for 40 s, and 72 °C for 45 s; followed by 72 °C for 10 min
	Reverse	TAGCTGGTCCTCTGCTGTCC	
