# Supplementary material for: Associations of tumor necrosis factor alpha genetic variants with metabolic syndrome and type 2 diabetes mellitus in a Thai population
Source: PLoS One. 2026 Apr 2;21(4):e0346147. doi: 10.1371/journal.pone.0346147 (PMC13046163; doi:10.1371/journal.pone.0346147)
Supplement: S2 Table — (PDF) [file pone.0346147.s002.pdf]

S2 Table. Tumor necrosis factor alpha variants, genotypes, minor allele frequency, and Hardy–Weinberg equilibrium test stratified by metabolic syndrome status.

Variants	Frequency (%)	
	Non-MetS *n* = 421	MetS * *n* = 344
rs1800629 (G>A)		
G/G	383 (91.0)	282(82.0)
G/A	36 (8.6)	59 (17.2)
A/A	2 (0.4)	3 (0.8)
MAF (%)	4.8	9.5
P value †	0.24	1.00
rs361525 (G>A)		
G/G	407 (96.7)	321 (93.3)
G/A	14 (3.3)	23 (6.7)
MAF (%)	1.7	3.3

Abbreviations: MAF, minor allele frequency; MetS, metabolic syndrome

* Metabolic syndrome was defined according to the 2009 International Diabetes Federation criteria.

† *P* value from Hardy–Weinberg equilibrium exact test
