# Supplementary material for: Associations of tumor necrosis factor alpha genetic variants with metabolic syndrome and type 2 diabetes mellitus in a Thai population
Source: PLoS One. 2026 Apr 2;21(4):e0346147. doi: 10.1371/journal.pone.0346147 (PMC13046163; doi:10.1371/journal.pone.0346147)
Supplement: S3 Table — (PDF) [file pone.0346147.s003.pdf]

S3 Table. Tumor necrosis factor alpha variants, genotypes, minor allele frequency, and Hardy–Weinberg equilibrium test stratified by type 2 diabetes mellitus status.

Variants	Frequency (%)	
	Non-T2DM *n* = 338	T2DM * *n* = 427
rs1800629 (G>A)		
G/G	311 (92.0)	354 (82.9)
G/A	25 (7.4)	70 (16.4)
A/A	2 (0.6)	3 (0.7)
MAF (%)	4.3	8.9
P value †	0.12	1.00
rs361525 (G>A)		
G/G	321 (95.0)	407 (95.3)
G/A	17 (5.0)	20 (4.7)
MAF (%)	2.5	2.4

Abbreviations: MAF, minor allele frequency; T2DM, type 2 diabetes mellitus

* Type 2 diabetes mellitus was diagnosed according to the American Diabetes Association criteria.

† *P* value from Hardy–Weinberg equilibrium exact test
