# Supplementary material for: Associations of tumor necrosis factor alpha genetic variants with metabolic syndrome and type 2 diabetes mellitus in a Thai population
Source: PLoS One. 2026 Apr 2;21(4):e0346147. doi: 10.1371/journal.pone.0346147 (PMC13046163; doi:10.1371/journal.pone.0346147)
Supplement: S4 Table — (PDF) [file pone.0346147.s004.pdf]

Supplementary Table 4 Logistic regression analysis of rs1800629 genotypes and their association with obesity, stratified by disease status (MetS and T2DM)

Disease status	Genotypes	Odds ratios ‡	P-values
Non-Mets	G/G	ref	-
	G/A	1.26	0.52
	A/A	NA	0.98
Mets*	G/G	ref	-
	G/A	0.30	0.59
	A/A	12.98	0.99
	G/G	ref	-
Non-T2DM	G/A	0.93	0.86
	A/A	NA	0.98
	G/G	ref	-
T2DM†	G/A	0.79	0.51
	A/A	13.69	0.99

Abbreviations: MetS, metabolic syndrome; T2DM, type 2 diabetes mellitus

* Metabolic syndrome was defined according to the 2009 International Diabetes Federation criteria.

† Type 2 diabetes (T2DM) was defined according to the American Diabetes Association (ADA) definition.

‡ Adjusted by age, sex and BMI. Odds ratios are shown as NA where estimates were not reliable due to sparse genotype counts.
